# Relationships among Inflammatory Biomarkers and Objectively Assessed Physical Activity and Sleep during and after Chemotherapy for Gynecologic Malignancies

**DOI:** 10.3390/cancers15153882

**Published:** 2023-07-30

**Authors:** Danielle B. Tometich, Aasha I. Hoogland, Brent J. Small, Michelle C. Janelsins, Crystal Bryant, Yvelise Rodriguez, Brian D. Gonzalez, Xiaoyin Li, Hailey W. Bulls, Brian W. James, Bianca Arboleda, Claudia Colon-Echevarria, Mary K. Townsend, Shelley S. Tworoger, Paulo Rodriguez, Laura B. Oswald, Julienne E. Bower, Sachin M. Apte, Robert M. Wenham, Hye Sook Chon, Mian M. Shahzad, Heather S. L. Jim

**Affiliations:** 1 Department of Health Outcomes and Behavior, Moffitt Cancer Center, Tampa, FL 33612, USA; 2 School of Aging Studies, University of South Florida, Tampa, FL 33620, USA; 3 Department of Surgery and Neuroscience, University of Rochester Medical Center, Rochester, NY 14642, USA; 4 Section of Palliative Care and Medical Ethics, Division of General Internal Medicine, Department of Medicine, University of Pittsburgh, Pittsburgh, PA 15260, USA; 5 Morsani College of Medicine, University of South Florida, Tampa, FL 33620, USA; 6 Department of Cancer Epidemiology, Moffitt Cancer Center, Tampa, FL 33612, USA; 7 Department of Immunology, Moffitt Cancer Center, Tampa, FL 33612, USA; 8 Department of Psychology, University of California Los Angeles, Los Angeles, CA 90095, USA; 9 Huntsman Cancer Institute, Department of Obstetrics and Gynecology, University of Utah, Salt Lake City, UT 84112, USA; 10 Department of Gynecologic Oncology, Moffitt Cancer Center, Tampa, FL 33612, USA

**Keywords:** gynecologic cancer, physical activity, sleep, inflammation

## Abstract

**Simple Summary:**

Physical inactivity and sleep problems are commonly reported by women going through chemotherapy for gynecologic cancer. Inflammation from cancer and its treatment might contribute to these issues, but existing findings are limited. We examined relationships between biomarkers of inflammation and data from wearable devices to objectively measure physical activity and sleep. We collected data from women with gynecologic cancer during chemotherapy and followed up with them for a year after completing chemotherapy. We also compared their results to women without cancer who were assessed at similar time intervals. We found that women with cancer were less active and had more sleep problems than controls even a year after completing chemotherapy. Greater inflammation was also related to less physical activity and more sleep problems. Future research should test whether interventions aimed at reducing inflammation can help women with cancer to be more active and have fewer sleep problems.

**Abstract:**

Little is known regarding associations between inflammatory biomarkers and objectively measured physical activity and sleep during and after chemotherapy for gynecologic cancer; thus, we conducted a longitudinal study to address this gap. Women with gynecologic cancer (patients) and non-cancer controls (controls) completed assessments before chemotherapy cycles 1, 3, and 6 (controls assessed contemporaneously), as well as at 6- and 12-month follow-ups. Physical activity and sleep were measured using wrist-worn actigraphs and sleep diaries, and blood was drawn to quantify circulating levels of inflammatory markers. Linear and quadratic random-effects mixed models and random-effects fluctuation mixed models were used to examine physical activity and sleep over time, as well as the associations with inflammatory biomarkers. On average, patients (*n* = 97) and controls (*n* = 104) were 62 and 58 years old, respectively. Compared to controls, patients were less active, more sedentary, had more time awake after sleep onset, and had lower sleep efficiency (*p*-values < 0.05). Across groups, higher levels of TNF-α were associated with more sedentary time and less efficient sleep (*p*-values ≤ 0.05). Higher levels of IL-1β, TNF-α, and IL-6 were associated with lower levels of light physical activity (*p*-values < 0.05). Associations between inflammatory biomarkers, physical activity, and sleep did not differ between patients and controls. Given these results, we speculate that inflammation may contribute to less physical activity and more sleep problems that persist even 12 months after completing chemotherapy.

## 1. Introduction

Over 1.4 million people in the United States are living with gynecologic cancers, including primary cancers of the ovary, uterus, and cervix [1]. Survival rates are improving for ovarian cancer but have remained relatively stagnant for uterine and cervical cancer for the past 50 years [1,2]. Currently, 5-year relative survival rates for gynecologic cancer range from 49% for ovarian to 81% for uterine cancer. Standard of care treatment includes surgery and chemotherapy for locally advanced ovarian cancer, surgery and/or combination chemotherapy/radiation for cervical cancers, and surgery followed by observation or chemotherapy and/or radiation for uterine cancers [1,3,4]. Although systemic chemotherapy treatment is often necessary for effective treatment, chemotherapy can result in distressing side effects that interfere with quality of life [5,6,7,8,9].

Inflammation is a potential mechanism to explain the relationship of cancer and its treatment with well-established ‘sickness behaviors’ associated with cancer, such as reduced physical activity and changes in sleep patterns [10,11]. Sleep disturbance reportedly affects up to 80% of people with gynecologic cancer during treatment [12], and prior research has found evidence for inflammation-associated sleep disturbance lasting at least one year after treatment for patients with ovarian cancer [13]. Physical activity can also be affected in those with gynecologic cancer, as fewer than 20% of ovarian cancer survivors report meeting recommendations for 150 min of moderate to strenuous physical activity per week [5]. Existing research on physical activity, sleep, and/or inflammation among individuals with gynecologic cancers is limited by the use of self-reported measures of physical activity and sleep [7,12,13,14,15], few assessments during chemotherapy [13,15] or short-term follow-ups of a few weeks up to four months after treatment [12,16], and/or a lack of control comparison [7,12,13,14,15,16]. There is currently a paucity of research designed to examine associations between inflammation, sleep, and physical activity during and following chemotherapy for gynecologic cancer. Collecting this information will inform biobehavioral symptom management interventions.

The goal of this study was to examine longitudinal relationships between circulating biomarkers of inflammation (i.e., IL-10, IL-1β, TNF-α, IL-6, IL-1Ra, TNFR1, TNFR2, and CRP) and objectively measured physical activity and sleep in individuals with gynecologic cancer during and after chemotherapy (i.e., patients) as compared to frequency age-matched individuals without cancer (i.e., controls), assessed contemporaneously. We hypothesized that (1) patients would have worse sleep and less physical activity than controls before, during, and after chemotherapy; (2) participants with higher levels of inflammation would report worse sleep and less physical activity than those with lower levels of inflammation (between-person effects); (3) at times when participants had higher levels of inflammation than their own average, they would also report worse sleep and less physical activity (within-person effects). We also explored differences in relationships among circulating biomarkers of inflammation and sleep and physical activity by group.

## 2. Materials and Methods

### 2.1. Participants

The study methodology has been described in detail elsewhere [17,18,19]. Briefly, participants were recruited between August 2013 and July 2018 prior to starting a new chemotherapy regimen for gynecologic malignancies at the Moffitt Cancer Center. Inclusion criteria were (1) 18–89 years of age; (2) able to speak and read English; (3) diagnosed with a gynecologic malignancy (e.g., ovarian, endometrial, uterine, cervical, vulvar, fallopian tube, or peritoneal); (4) scheduled to start intravenous or intraperitoneal chemotherapy; (5) no current or prior history of immune-related diseases (e.g., HIV, rheumatoid arthritis, systemic lupus erythematosus); (6) no documented psychiatric, sleep, or neurological disorders that could interfere with study participation (e.g., psychosis, sleep apnea, dementia); (7) no receipt of chemotherapy or radiation in the month prior to enrollment; (8) not pregnant; and (9) able to provide informed consent. Eligibility criteria for controls were the same apart from the presence of a gynecologic malignancy and chemotherapy. Additionally, controls were required not to have a history of any form of cancer except non-melanoma skin cancer, and they had to be within five years of age of the patient participant to whom they were being matched. This study was approved by the University of South Florida Institutional Review Board. 

Recruitment procedures for patients included physician referral, screening of clinic schedules, and in-person or telephone screening by a trained research coordinator to determine eligibility and obtain informed consent. Controls were recruited from a contact list from a national marketing company. Patients who consented to participate completed assessments at eight timepoints: pre-chemotherapy cycle 1 (i.e., 1 week before beginning treatment) and post-chemotherapy cycle 1 (i.e., 1 week after first treatment); pre- and post-chemotherapy cycle 3 (i.e., 1 week before and 1 week after cycle 3); pre- and post-chemotherapy cycle 6 (i.e., 1 week before and 1 week after cycle 6); 6-month follow-up (i.e., 1 week 6 months after cycle 6); and 12-month follow-up (i.e., 1 week 12 months after cycle 6). Controls were assessed contemporaneously (i.e., two-week assessments where first week coincides with pre-chemotherapy cycle and second week with post-cycle, 6 weeks between first two and second two assessments, 9 weeks between second two and third two assessments, and one-week assessments at 6 and 12 months after third two assessments). Participants were compensated USD 25 per assessment after returning the survey and actigraph. The current project used data from five timepoints: pre-chemotherapy cycle 1, pre-chemotherapy cycle 3, pre-chemotherapy cycle 6, 6-month follow-up, and 12-month follow-up.

### 2.2. Measures

#### 2.2.1. Sociodemographic and Clinical Variables

Demographics were self-reported by participants at baseline (i.e., age, race, education, household income, comorbidities, and menopausal status). Medical record review for cases only was conducted for clinical data (i.e., cancer type, cancer stage, prior lines of chemotherapy).

#### 2.2.2. Physical Activity and Sleep

Wrist-worn actigraph devices (ActiGraph GT3X, MiniMitter, Bend, OR, USA) were provided to participants, along with instructions to continuously wear the device on their non-dominant wrist for each assessment period (1 week per assessment). Prior research has found actigraphy to provide reliable and valid data for the estimation of sleep and physical activity in cancer populations [20,21,22]. Participants also completed daily sleep diaries of bedtimes and rising times. Sleep diary data were integrated with sustained periods of inactivity measured by actigraphy, and Cole/Kripke scoring algorithms [23] were used to estimate sleep efficiency (i.e., percentage of time in bed spent asleep) and the amount of time (in hours) awake after sleep onset. Data were downloaded via the ActiLife software (version 6.13.4, ActiGraph, Pensacola, FL) and raw data were exported to GGIR (version 2.8-2) in R (version 3.6.3) for processing. Criteria for data inclusion were at least 50% wear time and 3 valid wear days [24]. Outliers were reviewed and data were excluded if sleep time or activity appeared inconsistent with actigrams (i.e., graphs that showed physical activity over the entire wear period of the assessment for each participant). This procedure excluded data from 8 assessments (i.e., one patient and one control at baseline, one patient at pre-cycle 3, one control at the assessment corresponding to pre-cycle 6, one control at 6-month follow-up, and one patient and two controls at 12-month follow-up). For physical activity, movement over one-minute epochs was used to measure activity. After data processing, the daily activity level based on Euclidean Norms Minus One (ENMO) and the duration of each activity level were obtained in minutes and converted into hours. Thresholds for activity levels were <40 ENMO for sedentary behavior, 40–99 ENMO for light activity, and ≥100 ENMO for moderate-to-vigorous physical activity (MVPA) [20,21]. For sleep, data were excluded if the average sleep time was <3 or >12 h per night as these values were likely errors [25]. This excluded data from 3 assessments (i.e., one control at baseline, and one patient and one control at 12-month follow-up).

#### 2.2.3. Biomarkers of Inflammation

Participants provided blood samples at five timepoints: baseline (i.e., before starting chemotherapy for patients), before chemotherapy cycles 3 and 6 (and similar time intervals for controls), and again at 6 and 12 months after patients had completed chemotherapy. Each blood sample was evaluated for the presence of circulating markers of inflammation, which included interleukin 10 (IL-10), interleukin 1β (IL-1β), tumor necrosis factor α (TNF-α), interleukin 6 (IL-6), interleukin 1 receptor antagonist (IL-1Ra), tumor necrosis factor receptor 1 (TNFR1), tumor necrosis factor receptor 2 (TNFR2), and c-reactive protein (CRP). These inflammatory biomarkers were selected because previous studies have shown that they are associated with physical activity and sleep, they are relatively stable over time, and they are readily detectable using existing laboratory methodologies [10,11,13]. All blood samples were sent to the Cancer Control and Psychoneuroimmunology Lab at the University of Rochester for analysis. All samples were assayed in one run on a multiplexed cytokine bead assay (i.e., IL-10, IL-1β, TNF-α [HSTCMAG-28SK-04], TNFR1, TNFR2 [HSCRMAG-32K-02], IL-6, and IL-1Ra [HCYTOMAG-60K-01]) or using enzyme-linked immunosorbent assays (i.e., CRP; R&D Systems Human Quantikine ELISA; Minneapolis, MN, DCRP00; R&D Systems Human Quantikine ELISA; Minneapolis, MN, DCRP00) per the manufacturer’s instructions. The same lot was used for all kits. The median concentrated was taken from 50 beads per well for Luminex, and the average was taken from duplicates for ELISA. All data and internal controls were inspected for a CV < 20%, with all kits run with a standard curve with an r^2^ > 0.98. The same plate was used for all sample collections from the same participant. The lower limits of detection of the assays, with sample dilution taken into account, were IL-10 = 0.30; IL-1β = 0.14; IL-6 = 0.04; TNFα = 0.08; IL-1Rα = 7.41; TNFR1 = 10.60; TNFR2 = 10.18; CRP = 5.0 pg/mL.

### 2.3. Data Analyses

Circulating markers of inflammation with estimates below the lower limit of detection were divided by 1.4, and indeterminate inflammatory biomarker concentrations were set to missing. Inflammatory biomarker values that were three standard deviations from the sample mean for each group (cases and controls) were set to missing. Raw inflammatory biomarkers with non-normal distributions (i.e., IL-6, IL-1Ra) were natural log-transformed to normalize their distributions. All cytokines that were not natural log-transformed (i.e., IL-10, IL-1β, TNF-α, TNFR1, TNFR2, CRP) were mean-centered across all participants to facilitate interpretation.

Participants with non-missing data for at least one biomarker and actigraphy data at one or more timepoints were included in analyses. Sociodemographic and clinical characteristics of the sample were described using means, standard deviations, frequencies, and percentages. Independent-sample t-tests, chi-square tests, and Fisher’s tests were used to evaluate differences in sociodemographic and clinical characteristics between groups (i.e., patients vs. controls). Independent-sample t-tests were also used to evaluate differences in physical activity and sleep between groups at each timepoint. Linear and quadratic random-effects mixed models were used to examine changes in physical activity and sleep over time between each group and within each group. Time was coded as the number of months since baseline. Random-effects fluctuation mixed models were used to examine associations of inflammatory biomarkers with physical activity and sleep aggregated over the five assessments [26]. Biomarkers of inflammation were included as between-person predictors (having an average level of inflammation that differed from other participants) and within-person predictors (having an average level of inflammation that differed from a participant’s own average). To determine whether associations differed between patients and controls, interactions between the group and fluctuations in inflammatory biomarkers with physical activity and sleep were also included. Significant interactions between the group and fluctuations in inflammatory biomarkers were further probed within each group using separate random-effects fluctuation models with the group effect removed. Sensitivity analyses were completed by excluding patients with metastatic disease and examining fluctuations in inflammatory biomarkers with physical activity and sleep. All statistical analyses were conducted using SAS version 9.4 (Cary, NC, USA).

## 3. Results

The sociodemographic and clinical characteristics of patients (*n* = 97) and controls (*n* = 104) are displayed in Table 1. Patients were older, more likely to be White, and less likely to have a college education or a household income of USD 40,000 or more than controls (*p*-values < 0.05). Patients also reported more comorbidities on average (*p* = 0.05) and were more likely to be post-menopausal than controls (*p* < 0.01). Because of these group differences, we included age, race, education, comorbidities, and menopausal status as covariates. Income was not included as a covariate because it was highly correlated with education (Spearman’s rho = 0.34, *p* < 0.0001). Among patients, cancer types included ovarian (51%), endometrial (36%), and other gynecologic malignancies (13%).

### 3.1. Group Differences in Biomarkers of Inflammation

Raw and log-transformed (Il-6 and IL-1Ra only) means and longitudinal changes in biomarkers of inflammation in this study are reported elsewhere [27]. Patients generally had higher circulating levels of inflammatory biomarkers than controls over time. The results of the random-effects mixed models also demonstrated group differences over time such that the levels of some inflammatory biomarkers (i.e., TNFR1, TNFR2, IL-1Ra, CRP) generally decreased in patients but remained unchanged in controls.

### 3.2. Group Differences in Physical Activity and Sleep

Raw means for physical activity and sleep are presented in Table 2. Cross-sectional comparisons of physical activity (i.e., time in hours for MVPA, light activity, and sedentary time) and sleep (time in hours awake after sleep onset, sleep efficiency) by group indicated that patients spent significantly less time each day engaging in both light activity and MVPA than controls at all timepoints (patients engaged in 17.4 to 37.2 fewer minutes of MVPA and 18.6 to 44.4 min of light activity per day than controls). Patients were also significantly more sedentary throughout chemotherapy and at the 12-month follow-up compared to controls (patients were sedentary for 16.2 to 30 more minutes per day than controls). Patients had significantly more time awake after sleep onset than controls at all timepoints (patients spent 16.2 to 30 more minutes awake after sleep onset per night than controls) and had lower sleep efficiency than controls throughout chemotherapy and at 12-month follow-up (patients had 3–6% less efficient sleep than controls).

**Figure 1:**
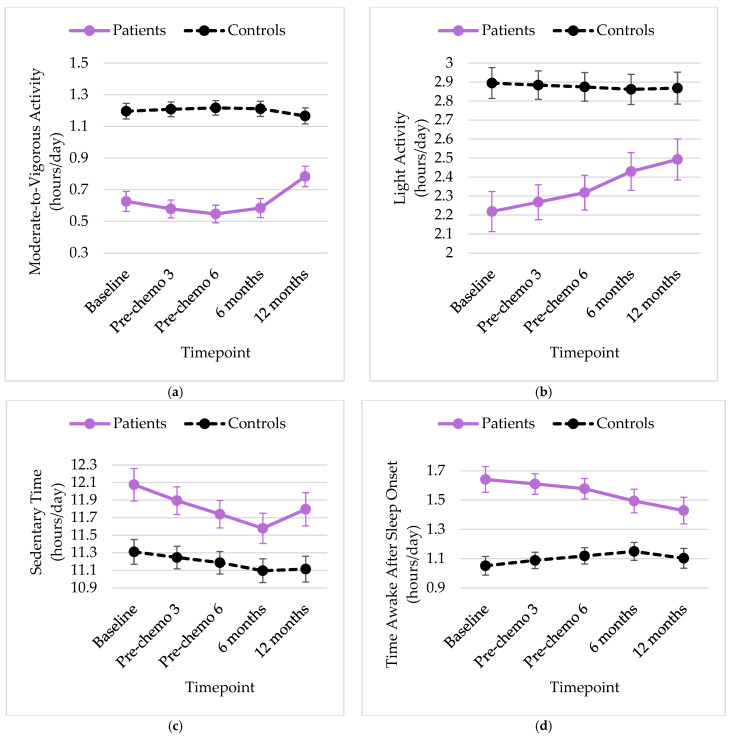

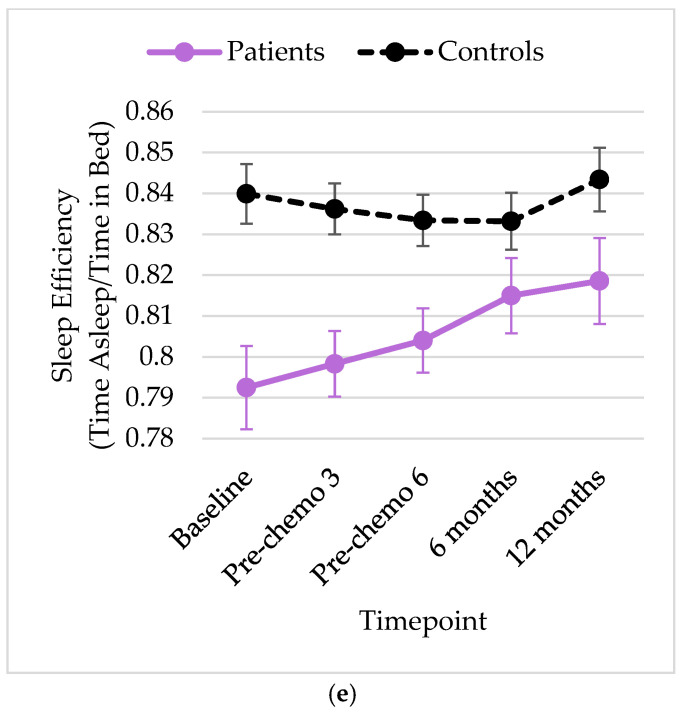
Least squares means over time by group for (**a**) moderate-to-vigorous activity hours per day, (**b**) light activity hours per day, (**c**) sedentary time hours per day, (**d**) average hours awake after sleep onset per night, (**e**) sleep efficiency, or proportion of time in bed spent sleeping.

Adjusted means of physical activity and sleep from the quadratic random-effects mixed models controlling for group (adjusted for age, race, education, comorbidities, and menopausal status) are displayed in Figure 1a–e. There was a significant quadratic change in MVPA for patients only, such that the number of hours spent in MVPA gradually decreased during chemotherapy and increased thereafter (from 0.63 h per day at baseline to 0.54 h before cycle 3 and 0.78 h at 12-month follow-up, *p* < 0.01). The results of the mixed models revealed significant interactions between group and time for light activity (*p* = 0.01) and time awake after sleep onset (*p* = 0.03). Specifically, the estimated hours per day of light activity increased over time for patients (from 2.22 h per day at baseline to 2.49 h at 12-month follow-up, *p* = 0.02) but remained unchanged for controls (2.86 to 2.89 h per day, *p* = 0.70). Although the interaction between group and time was significant for time awake after sleep onset in the entire sample, the effect of time was not significant for patients or controls when each group was examined separately (*p*-values > 0.05). There were no other significant changes in physical activity or sleep over time.

### 3.3. Longitudinal Relationships among Biomarkers of Inflammation and Physical Activity and Sleep

#### 3.3.1. Moderate-to-Vigorous Activity

The results of the random-effects fluctuation models are presented in Table 3. There was a significant interaction of within-person variance in IL-10 with group (*B* = 0.007, *p* = 0.04); there was no association between IL-10 and moderate-to-vigorous activity for controls (*B* = 0.002, *p* = 0.27). At times when patients had greater circulating IL-10, they tended to engage in less moderate-to-vigorous physical activity (*B* = −0.005, *p* = 0.05).

#### 3.3.2. Light Activity

Participants with higher levels of circulating IL-1β, TNF-α, and IL-6 (log-transformed) than their personal average spent significantly fewer hours in light activity each day (i.e., main effects of within-person variance in inflammation) (IL-1β: *B* = −0.04, *p* = 0.04; TNF-α: *B* = −0.02, *p* = 0.04; IL-6: *B* = −0.08, *p* = 0.02). There was also a significant interaction of within-person variance in IL-1β by group (*B* = 0.06, *p* = 0.04), such that there was no association between IL-1β and light activity for patients (*B* = 0.02, *p* = 0.49), but when controls had greater circulating IL-1β, they were engaged in less light activity (*B* = −0.04, *p* = 0.02).

#### 3.3.3. Sedentary Time

Participants with higher levels of circulating TNF-α than other participants tended to be more sedentary each day (i.e., main effect of between-person variance in inflammation; *B* = 0.04, *p* = 0.05). Participants with higher levels of circulating IL-10 than their personal average tended to be more sedentary each day (i.e., main effect of within-person variance in inflammation; *B* = 0.01, *p* = 0.05). There was also a trend for an interaction of within-person variance in CRP by group (*B* = 0.09, *p* = 0.05); however, this association of sedentary time with CRP was not significant when examined in patients (*B* = 0.03, *p* = 0.19) and controls separately (*B* = −0.005, *p* = 0.88).

#### 3.3.4. Wake after Sleep Onset

There were no significant associations between circulating markers of inflammation and time awake after sleep onset.

#### 3.3.5. Sleep Efficiency

Participants with higher levels of circulating TNF-α than their personal average also tended to have less efficient sleep (i.e., main effect of within-person variance in inflammation; *B =* −0.002, *p* = 0.05). There was also a significant interaction of between-person levels of IL-1Ra (log-transformed) and group on sleep efficiency (*B* = 0.02, *p* = 0.02); however, the association of IL-1Ra and sleep efficiency was not significant when examined in patients (*B* = 0.008, *p* = 0.17) and controls separately (*B* = −0.001, *p* = 0.64).

#### 3.3.6. Sensitivity Analyses

After excluding 16 patient participants with metastatic disease, the effects were similar, with a few exceptions (see Supplemental Table S1). For light activity, the main effect of group was no longer significant in the model with IL-1Ra (log-transformed; *B* = −0.25, *p* = 0.30), and the interaction effect of group and within-person levels of IL-1β was no longer significant (*B* = 0.03, *p* = 0.35). For sleep efficiency, there was an additional trend for a main effect of group in the model with IL-1β (*B* = −0.04, *p* = 0.05).

## 4. Discussion

Changes to physical activity and sleep are commonly reported by people with gynecologic cancer during chemotherapy [12,13], yet there is limited research on the objective measurement of these sickness behaviors and associations with inflammation during and following chemotherapy. To our knowledge, this is the first longitudinal study with a control group to examine inflammatory markers and objectively measured physical activity and sleep during and following chemotherapy for gynecologic cancer. Our findings provide evidence for affected physical activity and sleep during chemotherapy. Additionally, when participants had higher circulating levels of inflammatory cytokines relative to their own average, they also demonstrated greater sickness behaviors in the form of reduced physical activity and sleep efficiency.

Patients were overall less active and had more sleep problems than controls. Compared to controls, patients engaged in less light and moderate-to-vigorous physical activity at all timepoints, and patients were more sedentary during chemotherapy and at 12-month follow-up. Patients increased their activity over time, demonstrating some recovery of activity following chemotherapy; however, patient activity levels remained affected even at 12-month follow-up. Compared to controls, patients also had more time awake after sleep onset at all timepoints and lower sleep efficiency during chemotherapy. These findings are consistent with prior research showing that reduced physical activity and sleep problems are common and can be long-term side effects of chemotherapy [5,7,12,13,14,15]. Our findings extend prior research by showing these associations using objectively measured physical activity and sleep, and by demonstrating changes in physical activity and sleep over the course of six chemotherapy cycles (worsening) as well as 6- and 12-month follow-ups (improving) among patients with gynecologic cancer.

We found that several inflammatory markers were significantly associated with physical activity and sleep. When participants had higher circulating levels of IL-1β, TNF-α, and IL-6 than their usual average, they had lower light physical activity. The associations for TNF-α and IL-6 were similar for both cases and controls, suggesting common underlying relationships between inflammation and both activity and sleep. Further, participants who had higher circulating levels of TNF-α also were more sedentary. When participants had higher TNF-α than their own average, they also had less efficient sleep. IL-6, TNF-α, and IL-1β are proinflammatory cytokines [28]. Prior research has found that TNF-α is essential for the sleep–wake cycle, and both elevations and decrements can disrupt the homeostasis necessary for efficient sleep [11,29]. These results suggest that greater inflammation is associated with less light physical activity, more sedentary behavior, and less efficient sleep.

Interestingly, higher IL-10, which has anti-inflammatory properties, was previously found to be associated with less sedentary time and greater physical activity in a healthy sample without cancer [30]. However, in our study, when participants had higher IL-10, they were more sedentary and engaged in less moderate-to-vigorous physical activity. The previous study enrolled both men and women under the age of 55 years, with a mean age of 32, while our study had a much older population on average (60 years) and comprised only females. It is possible that there may be differences in associations in older groups, particularly postmenopausal women, who tend to have higher levels of inflammation [31]. There is also emerging evidence that IL-10 can be ‘non-classical’ and pro-inflammatory in the context of cancer [32]. Future research should evaluate potential differences in these associations across sex and age and determine the impact of cancer on functional changes in IL-10 action.

Strengths of our study include the unique contribution of objectively measured physical activity and sleep during chemotherapy, with a full one year of follow-up, and a comparison to controls. The inclusion of inflammatory biomarkers and associations with objective activity and sleep measurements are also strengths. However, our study also had limitations. The majority of participants were White, with middle to upper-level socioeconomic status, and the study was conducted at one cancer center, which may limit the generalizability of the findings to other populations. Prior research has found disparities in gynecological morbidity and mortality outcomes between Black and White women in the United States, and the likely main source of this disparity is inadequate access to screening, HPV vaccination, and cancer treatment [33]. To enhance the generalizability and investigate potential disparities in inflammation, sleep, and physical activity, multiple recruitment sites and community networking are recommended for future research [34]. There were also significant group differences in a few sample characteristics (i.e., education, menopausal status), and this limitation was addressed by including covariates in the analyses. Income is another potential confounding variable; however, income and education were highly correlated, and including both as covariates would have resulted in multicollinearity. A future project with a larger sample may be better able to control for additional confounders such as income, as well as variables that were unmeasured in the current study (e.g., body mass index, waist-to-hip ratio).

The findings point to several opportunities for future research. Interventions aimed at reducing inflammation, such as an anti-inflammatory diet or medications (such as bupropion), potentially could address sickness behaviors [35,36,37]. In addition, randomized trials of health behavior change interventions such as exercise and cognitive–behavioral therapy for insomnia have shown promise in reducing markers of inflammation among healthy middle-aged and older adults [38,39] and cancer survivors [40,41]. Thus, there may be bidirectional effects in the relationship between inflammation and sickness behaviors, and an intervention focused on one may have a cascading positive impact. Since many associations were similar between cases and non-cases, this suggests that interventions addressing sickness behaviors or reducing inflammation may work in both populations. For example, TNF-α inhibitors have been used to treat multiple auto-immune diseases [42], which also exhibit similar sickness behaviors to those seen in cancer patients. Future research should identify the health behaviors and specific inflammatory factors with the strongest effects, the factors most amenable to long-term change, and which interventions work best for which patients at which timepoints in the trajectory from treatment to survivorship. Future research may also improve the generalizability by recruiting diverse samples and utilizing multiple sites and community partnerships.

## 5. Conclusions

In conclusion, this study is among the first to examine the associations between inflammatory markers and objectively measured physical activity and sleep during and following chemotherapy for gynecologic cancer. The findings suggest that greater inflammation is associated with less light physical activity, more sedentary behavior, and more sleep problems in those with and without cancer, and that patients are less active and have more sleep problems than controls, possibly due to their higher inflammation levels. Clinical implications include a need to examine anti-inflammatory health behavior change interventions or drugs among people with gynecologic cancer to address patient-reported outcomes.

## Figures and Tables

**Table 1:** Participant characteristics.

Variable	Patients (*n* = 97)	Controls (*n* = 104)	*p*-Values
Age: M (SD)	61.62 (10.07)	58.38 (12.44)	0.05
Race: *n* (%) White	92 (96)	92 (89)	0.05
Education: *n* (%) college graduate	33 (34)	74 (71)	<0.0001
Income: *n* (%) USD 40,000 or more	51 (68)	73 (81)	0.05
Comorbidities: M (SD, range)	2.40 (0.85, 2–7)	2.19 (0.53, 2–5)	0.05
Menopausal status: *n* (%)			<0.01
Pre-menopausal	10 (11)	26 (25)	
Post-menopausal	84 (89)	78 (75)	
Cancer Type: *n* (%)		-	-
Cervical	1 (1)		
Fallopian	3 (3)		
Ovarian	49 (51)		
Vulvar	1 (1)		
Endometrial	35 (36)		
Peritoneal	5 (5)		
Other	2 (2)		
Stage: *n* (%)		-	-
1	18 (20)		
2	9 (10)		
3	48 (53)		
4	16 (18)		
Prior lines of chemotherapy: *n* (%) 3 or more	9 (9)	-	-

**Table 2:** Physical activity and sleep raw means.

	Baseline		Pre-Chemo 3		Pre-Chemo 6		6 Month Follow-Up		12 Month Follow-Up	
	Patients	Controls	Patients	Controls	Patients	Controls	Patients	Controls	Patients	Controls
**Physical Activity**										
MVPA ^a^	**0.55 (0.42)**	**1.17 (0.52)**	**0.74 (0.52)**	**1.29 (0.6)**	**0.64 (0.48)**	**1.15 (0.53)**	**0.85 (0.5)**	**1.14 (0.53)**	**0.71 (0.49)**	**1.12 (0.52)**
Light Activity ^b^	**2.1 (0.66)**	**2.84 (0.82)**	**2.34 (0.8)**	**3.04 (0.84)**	**2.21 (0.84)**	**2.82 (0.86)**	**2.6 (0.62)**	**2.91 (0.82)**	**2.38 (0.81)**	**2.9 (0.88)**
Sedentary Time ^c^	11.88 (1.27)	11.45 (1.38)	**11.68 (1.45)**	**11.2 (1.5)**	11.73 (1.42)	11.32 (1.34)	11.26 (1.51)	11.14 (1.24)	**11.79 (1.57)**	**11.24 (1.22)**
**Sleep Measures**										
WASO ^d^	**1.6 (0.76)**	**1.1 (0.56)**	**1.56 (0.78)**	**1.21 (0.73)**	**1.65 (0.95)**	**1.29 (0.73)**	**1.39 (0.75)**	**1.12 (0.64)**	**1.46 (0.77)**	**1.07 (0.62)**
Sleep Efficiency ^e^	**0.79 (0.09)**	**0.85 (0.06)**	**0.8 (0.08)**	**0.83 (0.08)**	**0.8 (0.09)**	**0.83 (0.08)**	0.82 (0.08)	0.84 (0.07)	**0.81 (0.08)**	**0.84 (0.08)**

^a^ MVPA = moderate-to-vigorous physical activity in hours per day. ^b^ Light activity in hours per day. ^c^ Sedentary time in hours per day. ^d^ WASO = wake after sleep onset in hours per night. ^e^ Sleep efficiency, or proportion of time in bed spent asleep. Significant group differences are in bold.

**Table 3:** Associations of fluctuations in biomarkers of inflammation with sleep and physical activity among patients with gynecologic cancer treated with chemotherapy and noncancer controls.

Variable	Moderate to Vigorous Activity							
	IL-10	IL-1β	TNF-α	TNFR1	TNFR2	CRP	IL-6^	IL-1Ra^
Intercept	1.76 ***	1.84 ***	1.85 ***	1.72 ***	1.86 ***	1.90 ***	1.81 ***	1.75 ***
Group	−0.46 ***	−0.70 ***	−0.73 ***	−0.52 **	−0.68 ***	−0.61 ***	−0.57 ***	−0.59 ***
Between-person variance in cytokine	2.91 × 10^−3^	−6.91 × 10^−3^	−8.71 × 10^−3^	5.26 × 10^−4^	−8.00 × 10^−5^	−0.03	0.04	7.88 × 10^−3^
Within-person variance in cytokine	2.46 × 10^−3^	−1.66 × 10^−3^	−1.77 × 10^−3^	−2.00 × 10^−5^	1.90 × 10^−5^	1.45 × 10^−3^	−0.01	0.02
Group × between-person variance in cytokine	−5.37 × 10^−3^	0.04	0.01	−4.20 × 10^−4^	1.05 × 10^−4^	0.02	−0.02	0.02
Group × within-person variance in cytokine	7.24 × 10^−3^ *	7.34 × 10^−3^	−8.89 × 10^−3^	8.10 × 10^−5^	2.00 × 10^−5^	−7.70 × 10^−3^	−0.01	−0.04
**Variable**	**Light Activity**							
	**IL-10**	**IL-1β**	**TNF-α**	**TNFR1**	**TNFR2**	**CRP**	**IL-6^**	**IL-1Ra^**
Intercept	3.14 ***	3.19 ***	3.15 ***	2.79 ***	2.93 ***	3.20 ***	3.04 ***	3.05 ***
Group	−0.75 ***	−0.79 ***	−0.48	−0.05	−0.20	−0.51 **	−0.50 ***	−0.42 *
Between-person variance in cytokine	−6.05 × 10^−3^	−0.03	−0.02	1.12 × 10^−3^	4.00 × 10^−5^	−0.05	−0.07	−0.04
Within-person variance in cytokine	−6.55 × 10^−3^	−0.04 *	−0.02 *	−3.80 × 10^−4^	−1.10 × 10^−4^	−8.12 × 10^−3^	−0.08 *	−0.03
Group × between-person variance in cytokine	0.01	0.09	5.36 × 10^−3^	−2.44 × 10^−3^	−2.50 × 10^−4^	0.03	0.06	−3.37 × 10^−3^
Group × within-person variance in cytokine	5.51 × 10^−3^	0.06 *	3.78 × 10^−3^	−1.16 × 10^−3^	−9.00 × 10^−5^	−0.04	0.06	−2.12 × 10^−3^
**Variable**	**Sedentary Time**							
	**IL-10**	**IL-1β**	**TNF-α**	**TNFR1**	**TNFR2**	**CRP**	**IL-6^**	**IL-1Ra^**
Intercept	10.86 ***	10.81 ***	10.55 ***	11.26 ***	11.02 ***	10.72 ***	10.99 ***	10.97 ***
Group	0.63	0.91 *	1.28 *	7.05 × 10^−3^	0.37	0.29	0.42	0.28
Between-person variance in cytokine	−1.34 × 10^−3^	0.02	0.04 *	−1.91 × 10^−3^	−7.00 × 10^−5^	0.06	0.06	6.91 × 10^−4^
Within-person variance in cytokine	0.01 *	0.05	6.83 × 10^−3^	1.13 × 10^−3^	1.17 × 10^−4^	−0.01	0.04	7.44 × 10^−3^
Group × between-person variance in cytokine	−4.13 × 10^−3^	−0.12	−0.06	3.20 × 10^−3^	1.48 × 10^−4^	0.02	3.76 × 10^−3^	0.05
Group × within-person variance in cytokine	8.95 × 10^−4^	−0.02	2.26 × 10^−4^	−1.96 × 10^−3^	−6.60 × 10^−4^	0.09 *	0.05	0.15
**Variable**	**Wake after Sleep Onset**							
	**IL-10**	**IL-1β**	**TNF-α**	**TNFR1**	**TNFR2**	**CRP**	**IL-6^**	**IL-1Ra^**
Intercept	1.50 ***	1.46 ***	1.38 ***	1.42 ***	1.45 ***	1.35 ***	1.49 ***	1.50 ***
Group	0.49 **	0.53 **	0.59 **	0.51 **	0.42	0.51 ***	0.44 ***	0.68 ***
Between-person variance in cytokine	−2.00 × 10^−4^	1.99 × 10^−4^	9.05 × 10^−3^	3.39 × 10^−4^	7.30 × 10^−5^	0.03	−8.63 × 10^−3^	−0.01
Within-person variance in cytokine	−2.96 × 10^−3^	−1.22 × 10^−3^	−6.25 × 10^−3^	−4.10 × 10^−4^	−6.00 × 10^−5^	1.72 × 10^−3^	−0.01	−0.05
Group × between-person variance in cytokine	−2.88 × 10^−3^	−0.03	−0.01	−5.30 × 10^−4^	−9.88 × 10^−6^	−0.03	−0.14	−0.12
Group × within-person variance in cytokine	−1.88 × 10^−3^	0.03	8.23 × 10^−3^	4.00 × 10^−5^	1.35 × 10^−4^	−1.04 × 10^−3^	0.04	−0.01
**Variable**	**Sleep Efficiency**							
	**IL-10**	**IL-1β**	**TNF-α**	**TNFR1**	**TNFR2**	**CRP**	**IL-6^**	**IL-1Ra^**
Intercept	0.80 ***	0.80 ***	0.82 ***	0.81 ***	0.81 ***	0.84 ***	0.81 ***	0.81 ***
Group	−0.04 *	−0.03	−0.05 *	−0.03	−0.03	−0.04 *	−0.04 **	−0.08 ***
Between-person variance in cytokine	4.70 × 10^−4^	1.03 × 10^−3^	−1.34 × 10^−3^	−2.00 × 10^−5^	−2.00 × 10^−5^	−4.96 × 10^−3^	3.93 × 10^−3^	−1.02 × 10^−3^
Within-person variance in cytokine	−5.20 × 10^−4^	−3.78 × 10^−3^	−1.92 × 10^−3^ *	9.00 × 10^−5^	1.90 × 10^−5^	−1.97 × 10^−3^	−7.81 × 10^−3^	−2.32 × 10^−3^
Group × between-person variance in cytokine	2.04 × 10^−4^	9.06 × 10^−4^	1.53 × 10^−3^	7.50 × 10^−6^	1.54 × 10^−6^	3.90 × 10^−3^	9.58 × 10^−3^	0.02 *
Group × within-person variance in cytokine	8.70 × 10^−4^	3.99 × 10^−3^	5.50 × 10^−4^	−1.40 × 10^−4^	−3.00 × 10^−5^	3.41 × 10^−3^	5.32 × 10^−3^	0.01

Analyses controlled for age, education, comorbidities, and menopausal status. IL-6^ and IL-1Ra^ were natural log-transformed. *** = *p* < 0.001; ** = *p* < 0.01; * = *p* < 0.05.

## Data Availability

The data presented in this study are available on request from the corresponding author. The data are not publicly available due to patient participant privacy.

## Supplementary Materials

The following supporting information can be downloaded at: https://www.mdpi.com/article/10.3390/cancers15153882/s1, Table S1: Sensitivity analyses: Associations of fluctuations in biomarkers of inflammation with sleep and physical activity among patients with gynecologic cancer treated with chemotherapy and noncancer controls excluding those with metastatic disease.
