# Supplementary material for: Relationships among Inflammatory Biomarkers and Objectively Assessed Physical Activity and Sleep during and after Chemotherapy for Gynecologic Malignancies
Source: Cancers (Basel). 2023 Jul 30;15(15):3882. doi: 10.3390/cancers15153882 (PMC10416903; doi:10.3390/cancers15153882)
Supplement: Supplementary file 1 [file cancers-15-03882-s001.zip › cancers-2455165-supplementary.pdf]

Supplemental Table S1. Sensitivity analyses: Associations of fluctuations in biomarkers of inflammation with sleep and physical activity among patients with gynecologic cancer treated with chemotherapy and noncancer controls excluding those with metastatic disease

	**Moderate to Vigorous Activity**							
**Variable**	**IL-10**	**IL-1b**	**TNF-alpha**	**TNFR1**	**TNFR2**	**CRP**	**IL6^**	**IL-1ra^**
Intercept	1.79***	1.85***	1.88***	1.75***	1.89***	1.93***	1.84***	1.78***
Group	-0.44***	-0.68***	-0.68***	-0.52**	-0.68***	-0.62***	-0.53***	-0.52***
Between-person variance in cytokine	2.99E-03	-6.69E-03	-8.56E-03	5.29E-04	-8.00E-05	-0.03	0.04	7.99E-03
Within-person variance in cytokine	2.46E-03	-1.65E-03	-1.77E-03	-2.00E-05	1.80E-05	1.38E-03	-0.01	0.02
Group x between-person variance in cytokine	-5.70E-03	0.04	0.01	-3.10E-04	9.00E-05	0.02	-0.05	4.62E-03
Group x within-person variance in cytokine	-8.14E-03*	6.46E-03	-9.87E-03	2.80E-05	-1.00E-05	-8.03E-03	-0.01	-0.06

	**Light Activity**							
**Variable**	**IL-10**	**IL-1b**	**TNF-alpha**	**TNFR1**	**TNFR2**	**CRP**	**IL6^**	**IL-1ra^**
Intercept	3.20***	3.23***	3.24***	2.90***	3.03***	3.29***	3.12***	3.12***
Group	-0.69***	-0.78***	-0.52	-0.13	-0.27	-0.54**	-0.50***	-0.25
Between-person variance in cytokine	-5.99E-03	-0.03	-0.02	1.03E-03	2.80E-05	-0.05	-0.07	-0.04
Within-person variance in cytokine	-6.55E-03	-0.04*	-0.02*	-3.90E-04	-1.10E-04	-8.26E-03	-0.08*	-0.03
Group x between-person variance in cytokine	0.01	0.09	7.37E-03	-2.06E-03	-2.00E-04	0.03	0.05	-0.06
Group x within-person variance in cytokine	-1.03E-03	0.03	1.63E-03	-1.77E-03	-3.00E-05	-0.04	0.05	-0.06

	**Sedentary Time**							
**Variable**	**IL-10**	**IL-1b**	**TNF-alpha**	**TNFR1**	**TNFR2**	**CRP**	**IL6^**	**IL-1ra^**
Intercept	10.65***	10.66***	10.35***	11.05***	10.79***	10.45***	10.76***	10.75***
Group	0.65	0.83*	1.31*	0.09	0.46	0.43	0.44	0.25
Between-person variance in cytokine	-1.41E-03	0.02	0.04*	-1.73E-03	-6.00E-05	0.06	0.06	4.19E-03
Within-person variance in cytokine	0.01*	0.05	6.81E-03	1.13E-03	1.16E-04	-0.01	0.04	7.43E-03
Group x between-person variance in cytokine	-1.65E-03	-0.07	-0.05	3.07E-03	1.33E-04	3.60E-03	0.21	0.08
Group x within-person variance in cytokine	2.25E-03	0.01	1.41E-03	-3.96E-03	-8.20E-04	0.09*	0.04	0.18

	**Hours Awake after Sleep Onset**							
**Variable**	**IL-10**	**IL-1b**	**TNF-alpha**	**TNFR1**	**TNFR2**	**CRP**	**IL6^**	**IL-1ra^**
Intercept	1.50***	1.46***	1.37***	1.41***	1.45***	1.34***	1.50***	1.49***
Group	0.50**	0.50**	0.59**	0.49**	0.38	0.53***	0.45***	0.73***
Between-person variance in cytokine	-1.80E-04	3.35E-04	8.87E-03	3.14E-04	6.50E-05	0.03	-8.01E-03	-0.01
Within-person variance in cytokine	-2.95E-03	-1.18E-03	-6.24E-03	-4.20E-04	-6.00E-05	1.76E-03	-0.01	-0.05
Group x between-person variance in cytokine	-3.40E-03	-0.03	-0.01	-4.20E-04	2.60E-05	-0.03	-0.22	-0.13
Group x within-person variance in cytokine	-3.23E-03	0.03	6.91E-03	-2.90E-04	1.21E-04	-1.78E-03	0.05	-0.03

Variable	Sleep Efficiency							
	IL-10	IL-1b	TNF-alpha	TNFR1	TNFR2	CRP	IL6^	IL-1ra^
Intercept	0.79***	0.80***	0.81***	0.81***	0.81***	0.83***	0.81***	0.81***
Group	-0.04*	-0.04*	-0.06*	-0.03	-0.03	-0.05*	-0.05**	-0.09***
Between-person variance in cytokine	4.53E-04	9.87E-04	-1.35E-03	-2.00E-05	-2.00E-05	-5.15E-03	3.82E-03	-1.29E-03
Within-person variance in cytokine	-5.20E-04	-3.78E-03	-1.92E-03*	1.90E-05	1.90E-05	-1.97E-03	-7.80E-03	-2.31E-03
Group x between-person variance in cytokine	3.57E-04	1.49E-03	1.96E-03	7.42E-06	5.71E-07	4.71E-03	0.02	0.02*
Group x within-person variance in cytokine	6.37E-04	4.24E-03	4.81E-04	-2.50E-04	-4.00E-05	3.42E-03	4.13E-03	0.01

Analyses controlled for age, education, comorbidities, and menopausal status.

^IL-6 and IL-1Ra were natural log-transformed.

*** = p<.001; ** = p<.01; * = p<.05
